# The development of CHAMP: a checklist for the appraisal of moderators and predictors

**DOI:** 10.1186/s12874-017-0451-0

**Published:** 2017-12-21

**Authors:** Ralph van Hoorn, Marcia Tummers, Andrew Booth, Ansgar Gerhardus, Eva Rehfuess, Daniel Hind, Patrick M. Bossuyt, Vivian Welch, Thomas P. A. Debray, Martin Underwood, Pim Cuijpers, Helena Kraemer, Gert Jan van der Wilt, Wietkse Kievit

**Affiliations:** 1 Radboud Institute for Health Sciences, Radboud university medical center, Geert Grooteplein 21, Nijmegen, 6525 EZ The Netherlands; 2 Health Economics and Decision Science (HEDS), School of Health and Related Research (ScHARR), University of Sheffield Regent Court, Sheffield, UK; 3 Department of Health Services Research, Institute for Public Health and Nursing Research, University of Bremen and Health Sciences Bremen, University of Bremen, Bremen, Germany; 4 Institute for Medical Information Processing, Biometry and Epidemiology; Pettenkofer School of Public Health, LMU Munich, Munich, Germany; 5 Clinical Trials Research Unit, University of Sheffield Regent Court, Sheffield, UK; 6 Department of Clinical Epidemiology, Biostatistics, and Bioinformatics, University of Amsterdam, Amsterdam, The Netherlands; 7 Bruyère Institute, Ottawa, Canada; 8 Julius Center for Health Sciences and Primary Care, University Medical Center Utrecht, Utrecht University; Cochrane Netherlands, University Medical Center Utrecht, Utrecht, The Netherlands; 9 Warwick Clinical Trials Unit, Warwick Medical School, University of Warwick, Coventry, UK; 10 Department of Clinical, Neuro and Developmental Psychology, Amsterdam Public Health research institute, Vrije Universiteit Amsterdam, Amsterdam, The Netherlands; 11 Department of Psychiatry and Behavioral Sciences, Stanford University, Stanford, CA USA; 12 Donders Institute for Brain, Cognition and Behaviour, Radboud university medical center, Nijmegen, The Netherlands; 13 Institute for Health Sciences, Radboud university medical center, Nijmegen, The Netherlands

**Keywords:** Moderator, Predictor, Quality assessment, Subgroup, Personalized health care, Evidence based medicine, Delphi

## Abstract

**Background:**

Personalized healthcare relies on the identification of factors explaining why individuals respond differently to the same intervention. Analyses identifying such factors, so called predictors and moderators, have their own set of assumptions and limitations which, when violated, can result in misleading claims, and incorrect actions. The aim of this study was to develop a checklist for critically appraising the results of predictor and moderator analyses by combining recommendations from published guidelines and experts in the field.

**Methods:**

Candidate criteria for the checklist were retrieved through systematic searches of the literature. These criteria were evaluated for appropriateness using a Delphi procedure. Two Delphi rounds yielded a pilot checklist, which was tested on a set of papers included in a systematic review on reinforced home-based palliative care. The results of the pilot informed a third Delphi round, which served to finalize the checklist.

**Results:**

Forty-nine appraisal criteria were identified in the literature. Feedback was obtained from fourteen experts from (bio)statistics, epidemiology and other associated fields elicited via three Delphi rounds. Additional feedback from other researchers was collected in a pilot test. The final version of our checklist included seventeen criteria, covering the design (e.g. a priori plausibility), analysis (e.g. use of interaction tests) and results (e.g. complete reporting) of moderator and predictor analysis, together with the transferability of the results (e.g. clinical importance). There are criteria both for individual papers and for bodies of evidence.

**Conclusions:**

The proposed checklist can be used for critical appraisal of reported moderator and predictor effects, as assessed in randomized or non-randomized studies using individual participant or aggregate data. This checklist is accompanied by a user’s guide to facilitate implementation. Its future use across a wide variety of research domains and study types will provide insights about its usability and feasibility.

**Electronic supplementary material:**

The online version of this article (10.1186/s12874-017-0451-0) contains supplementary material, which is available to authorized users.

## Background

It is widely accepted that the evaluation of healthcare interventions should encompass not only overall effectiveness, but also the identification of factors that may influence effectiveness in individual patients [1]. Individuals receiving the same or a similar treatment may show widely differing responses due to differences in treatment dosage or administration, or differences in patient-level characteristics such as age and genetic makeup [2, 3]. Understanding of how patient-level characteristics influence treatment effects may increase the overall effectiveness of health technologies, help to avoid adverse events, and enhance overall patient satisfaction with the treatment(s) received [4–7]. Increasingly, it is recognized that substantial health benefits may be obtained by paying attention to such differences between individuals [8–10].

**Fig. 1:**
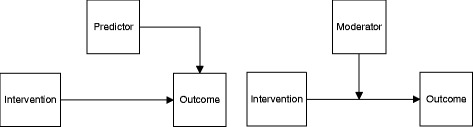
Schematic overview of two models containing a simple relationship between an input variable (e.g. intervention), an output variable (e.g. effect) and a predictor (left) or moderator (right)

Over the past few decades, several methods have been proposed for evaluating heterogeneity in treatment response. These methods typically distinguish between predictors and moderators of treatment response (Fig. 1) [11, 12].

Predictors are factors that are associated (either directly or indirectly) with a patient’s outcome, regardless of the intervention received. For instance, older patients may exhibit relapses of a disease more often than younger patients, making old age a *predictor* of relapses. Moderators are factors that allow estimation of an effect for a *specific* intervention for a group of patients with specific factors. The effect is brought about by a specific interaction with the intervention. This is the case when, for example, a relapse of a disease is better controlled with one treatment than with another, but the difference between treatments is smaller for younger patients. In that case, age is a moderator of treatment effect. In the literature, the terms ‘moderator’ and ‘predictor’ are occasionally used interchangeably (or other terms are used to describe their effects, such as effect modifier, determinant or interaction effect). Hence, it is important that the user first identifies whether the effect that is being appraised is actually a moderator, a predictor or other effect (e.g. an intermediate effect (mediator), or main effect).

In medical research, methods such as regression or subgroup analyses are often employed to test hypotheses concerning sources of heterogeneity. These methods can be conducted as secondary analysis within individual trials. Several assumptions and limitations are associated with these methods [10, 11]. Violating these assumptions and ignoring these limitations can result in questionable claims concerning the validity of treatment effects [13–16]. Critical appraisal is required before making clinical or policy decisions based on such information.

Most guidance on literature appraisal is aimed at valuing the primary outcome of a study; the overall validity, methods and other study properties that determine the relevance and credibility of the presented outcomes. Although several guidance documents exist to help researchers identify predictor and moderator variables [15, 17–20], their usefulness is often limited as they generally presume the user has more in depth knowledge of statistical methodologies. Moderator and predictor analyses are different from analyses related to the main outcome in multiple ways. They are more complicated, and since they are based on subpopulation of the main study population, they are associated with greater uncertainties. Existing guidance documents on predictor and moderator variables target specific study types (e.g. identification of effect modifiers in meta-analysis of published randomized trials) or fail to justify or clarify how items are to be defined or used. In addition, there is inconsistent usage of the “predictor” and “moderator” terms, further complicating the identification and critical appraisal of published intervention studies.

### Aim

The aim of our study was to create a uniform checklist for critically appraising the results of studies investigating factors of heterogeneity in treatment response. The checklist should be applicable in both randomized and non-randomized studies, and for both studies using individual patient data as aggregated data. The resultant checklist is intended for use in evaluating the validity of claims in studies for inclusion in health technology assessments, evaluating evidence for creating protocols in health care or constructing an evidence base for systematic reviews. To our knowledge, this is the first checklist to critically appraise reported moderators and predictors of treatment effect, with respect to their credibility, transferability and relevance for use.

## Methods

This study was conducted as part of the INTEGRATE-HTA project. INTEGRATE-HTA is an EU-funded project (http://www.integrate-hta.eu/) which aims to further refine methods of healthcare evaluation to take into account complexity. The heterogeneity of patients resulting in different treatment effects may be conceived as a source of complexity [21].

### Initial choices

In starting to develop the appraisal checklist, we defined the following criteria:

- Users should first be encouraged to use existing tools (see for instance those included in several systematic reviews [22, 23]) to appraise the overall risk of bias for the main effect in any given study. Appraisal of the moderator/predictor analysis is only to be pursued if the overall risk of bias is considered acceptable, as a lack thereof precludes adequate moderator/predictor analysis;
- The checklist should facilitate critical appraisal regarding claims about moderators or predictors described in a body of evidence, but also facilitate critical appraisal of individual studies;
- The checklist should not yield a summary or weighted score, as any weights would be arbitrary and hard to justify [24, 25]. Instead, users are invited to reach an overall, qualitative judgment in a structured manner;
- The checklist should consist of closed questions, with answering categories including ‘yes’, ‘no’, ‘don’t know’ and ‘not applicable’. The third option allows users to indicate that they do not consider themselves sufficiently qualified to judge the relevant item, or that the article does not report sufficient information to answer the question, while the fourth indicates that the item does not apply to the moderator or predictor being appraised.

### Procedure of checklist development

The development of the appraisal checklist consisted of four steps: (1) systematic searches for existing literature on moderator and predictor analysis to identify candidate criteria; (2) a Delphi procedure to select criteria that were considered most relevant for inclusion in the checklist; (3) pilot testing of the draft checklist; and (4) an evaluation of a modified checklist in a final Delphi round.

#### Step 1: Literature search

PubMed and Google Scholar were searched to identify candidate criteria for moderators and predictors of treatment effect, relying on the assumption that these two databases taken together cover most of the relevant literature in health and related fields. In PubMed, MeSH-terms (e.g. “Effect modifier, Epidemiologic”, “Randomized Controlled Trials as Topic”, “Moderators of treatment effects”, “moderators”, “subgroup”, “heterogeneity” and combinations thereof) were combined with keywords relating to appraisal (e.g. “critical appraisal”, “appraisal”, “guidance”, “methodology”, “quality assessment”). The same set of keywords was used to search in Google Scholar.

Key citations were identified and used to initiate additional searches based on their keywords and MeSH-terms, citation tracking and author searches. All search results were scanned for possible relevant content based on title and abstract (PubMed), or title and visible text snippets (Google Scholar) by one author (RvH).

Based on the selection of relevant papers, a list of candidate criteria for either moderator or predictor analysis was compiled. Duplicates were eliminated. Some criteria were slightly rephrased to produce a uniform answering format (e.g. transforming a statement into a polar question). Criteria were then classified so that they mapped to specific sections of a typical research article, thus improving the usability of the checklist.

#### Step 2: Delphi procedure

Thirty-seven experts were invited to determine which criteria of the list extracted in step 1 should be considered appropriate for the checklist. The experts were identified during the literature search and through our network, and included epidemiologists and (bio)statisticians from several European and North American countries.

A Delphi procedure was used to elicit the experts’ opinions in accordance with the Research ANd Development (RAND) Appropriateness Method manual [26]. In the first round, participants were asked to rate the appropriateness of individual criteria for inclusion in the checklist on an interval scale of 1 (not appropriate) to 9 (highly appropriate). Participants were also given the opportunity to propose re-formulations or additional criteria. In a second round, participants were asked to rate the same criteria (including rewordings or additions), excluding those criteria that had been agreed to be included or excluded in the first round. For each round, experts were invited by email and reminded up to two times. The Delphi rounds were conducted through online questionnaires.

Candidate criteria were removed from the list if an agreed appropriateness score of <4 was reached in any of the Delphi rounds according to the InterPercentile Range Adjusted for Symmetry (IPRAS)-method [26]. Criteria were included if they scored >6 and agreement had been reached according to the IPRAS method. The remaining criteria were considered inconclusive. The core research team from the INTEGRATE-HTA project (RvH, WK, MT and GJvdW) was tasked with handling the comments and suggested rewordings following each round, as well as the implementation of decisions to include or exclude appraisal criteria. Ultimately, the first two Delphi rounds resulted in a test version of the checklist containing the full set of consensual criteria.

#### Step 3: Pilot testing

The INTEGRATE-HTA project included a case-study which was used to demonstrate the methods described in the project [27]. Several researchers involved in this project appraised a set of papers with a pilot version of the checklist we named CHecklist for the Appraisal of Moderators and Predictors (CHAMP) (RvH, MT, WK, AB, AG, and CL). This test set consisted of twenty-two papers reporting on the effectiveness of reinforced models of home-based palliative care. [28–49] Comments and feedback were collected regarding the checklist as a whole and on individual criteria (e.g. concerning usability, clarity, or applicability). Furthermore, inter-rater agreement on extracted scores was calculated to determine whether the included criteria were used/interpreted similarly by different researchers. Subsequently, the checklist was revised based on these results and feedback from the researchers.

#### Step 4: Evaluation

In this final step, the new version of the checklist was presented to two participants of the Delphi procedure for early, in-depth feedback on the revisions. Based on their comments and feedback, adjustments were made and the revised checklist was presented (Delphi round 3) to the entire panel of experts who had participated in the previous two rounds of the Delphi procedure. The panel was asked whether they agreed with the content, form and design of the checklist, and was given the option of providing additional comments. Based on these comments, the checklist was finalized.

## Results

### Step 1: Literature search

During the literature search, five articles were identified as key citations [14, 15, 17, 50, 51]. These citations were used to further grow the number of search terms and to initiate citation chasing (forward/backward citation searches). Ultimately, forty-nine candidate criteria were identified for the appraisal of moderator/predictor analyses. As some of the criteria applied to a body of evidence (i.e. a systematic review or multiple related studies), these criteria were grouped in a separate category. Additional file 1 presents the complete list of criteria, their origin and changes throughout the development process of the checklist, as well as testing phase feedback and statistics.

**Fig. 2:**
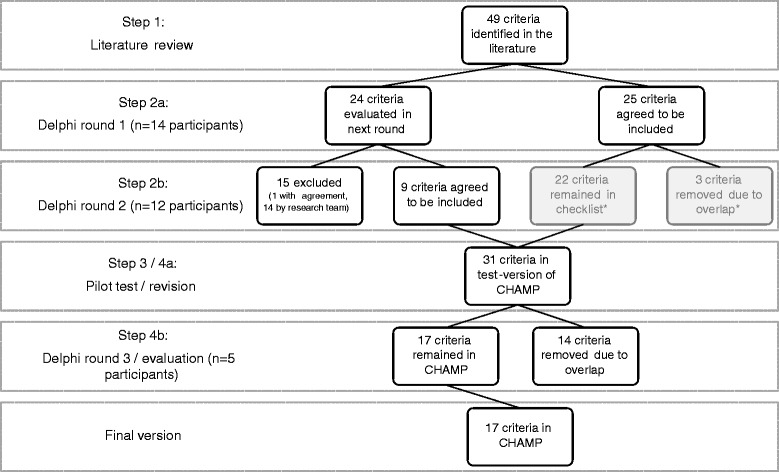
Process of inclusion and exclusion of appraisal criteria throughout the development procedure

Figure 2 is a flowchart of the entire procedure, outlining the number of appraisal criteria and experts involved in each step of the development of the checklist.

### Step 2: Delphi procedure

#### Delphi round 1

During the first Delphi round, the 49 criteria were rated by 14 experts (37 invited). Based on their assigned scores (mean score 6.9, range 6.1–8.1), 25 criteria were included. There was insufficient agreement on the remaining 24 criteria. Among these, five had a sufficient appropriateness score (mean 6.9, range 6.3–7.3), and 19 had an inconclusive appropriateness score (mean 5.3, range 4.6–5.9). These 24 criteria were re-evaluated in the second Delphi round. Suggestions for rewording and additional criteria were considered and changes made if considered appropriate.

#### Delphi round 2

Of the 14 experts, 12 provided feedback during the second Delphi round. Participants were also encouraged to assess potential overlap between the remaining criteria. Based on the results of this round, one criterion was removed following general agreement on low appropriateness (3.33). Seven criteria were selected for inclusion in the checklist (mean 6.9, range 6.1–7.7). Fourteen criteria were excluded based on low appropriateness and negative feedback. The remaining two criteria, which had appropriateness scores below the threshold but with no agreement, were put forward to the test phase. Rephrasing of criteria was performed based on feedback. This resulted in the merging of three criteria already accepted in round 1. At the end of the second Delphi round, the checklist consisted of 31 criteria. Of these, 20 criteria were applicable to individual studies, eight to systematic reviews and three to a set of related studies.

### Step 3: Pilot testing

Six researchers tested the 20 criteria that were deemed applicable to individual studies. This pilot checklist was tested on individual studies including RCTs, observational/cohort studies and qualitative research. The criteria intended to be used for systematic reviews and multiple related studies could not be tested here, as no systematic reviews or multiple related studies on reinforced models of home-based palliative care were found to cover the same moderator/predictor. The inter-rater agreement for most criteria included in the pilot test was limited, mainly due to differences in interpretation which resulted from insufficient clarification of single criteria. For example, users indicated difficulties in interpreting phrases such as ‘in the case of hypothesis testing’ (on what grounds should one decide whether this condition is fulfilled), or ‘low number of hypotheses’ (expecting the checklist to describe a specific threshold).

None of the criteria was excluded in this round. Some criteria were rephrased for improved ease of interpretation. In addition, a user’s guide was compiled to clarify how each criterion should be answered and to indicate possible consequences if specific criteria were not met.

### Step 4: Evaluation

Based on the feedback of two experts from the Delphi panel, the criteria specific for systematic reviews and the criteria addressing a body of evidence (*n* = 11) were placed in the same category and rephrased into five criteria covering a body of evidence. This step was taken as most criteria concerning systematic reviews were found to be interchangeable with those for multiple related studies. Due to overlap, another eight criteria were removed or described under the explanation of another criterion. This step resulted in a final version of the checklist containing 17 criteria in total, of which 14 were unique. Three criteria were included twice because they applied to individual studies as well as a body of evidence.

#### Delphi round 3

Five of the original 14 participants responded to the invitation for final feedback. A small number of adjustments were made to improve the introduction of the checklist and the user’s guide.

### The final checklist

Table 1 lists the 17 criteria in the final version of the checklist (Additional file 2). The final version also contains a short introduction and definitions. Furthermore, it contains references to appraisal checklists that might be used to determine the overall quality of a study, to be chosen at the users’ discretion. A guide is included, indicating the type of information that the user should look for within an article and explaining the rationale for each criterion to allow the user to better estimate its impact.

**Table 1:** Questions in the checklist for assessing moderators and predictors of treatment effects. Note that questions 10–12 are listed also as questions 13–15, as they are applicable both to individual studies and bodies of evidence covering the same moderator or predictor

Criteria for individual studies Design
1. A priori plausibility: was there sufficient empirical or theoretical support for the moderator or predictor that was examined? 2. Was the moderator or predictor specified a priori? 3. Was the moderator or predictor variable measured before the allocation or start of the intervention? 4. Was measurement of the moderator or predictor reliable and valid in the target population?
Analysis
5. In case of a moderator, was an interaction test used? 6. Was a limited number of moderators and predictors tested? 7. Was sample size adequate for the moderator or predictor analysis?
Results
8. Were results presented for all candidate moderators or predictors that were examined? 9. Did statistical tests or confidence intervals indicate that observed moderator or predictor effects were unlikely to be merely due to chance variation? 10. Was the moderator or predictor effect consistent with related moderators or predictors, or across related outcomes measured within the study?
Transferability
11. Were the setting and study population comparable to the setting and population in which the information would be used? 12. Is the moderator or predictor effect clinically important?
Criteria for bodies of evidence (systematic review or related sets of studies)
13. Was the moderator or predictor effect consistent with related moderators or predictors, or across related outcomes measured between the studies? 14. Were the setting and study population comparable to the setting and population in which the information would be used? 15. Is the moderator or predictor effect clinically important? 16. Was the moderator or predictor effect reasonably homogenous across studies? 17. Was the moderator or predictor measured similarly across the included studies, or was an adequate conversion performed?

## Discussion

The product of this study is a checklist which can be used to appraise claims concerning moderators or predictors in individual studies as well as bodies of evidence. The checklist is envisioned to be used by authors of systematic reviews investigating a specific (set of) moderator or predictor effects, or by researchers who need to identify relevant moderators or predictors to consider for clinical decision making. The checklist can be used by both experts and those less knowledgeable of moderator or predictor analysis. The checklist may also be used by journal editors and reviewers who aim to assess the methodological quality of studies assessing a moderator or predictor effect for a certain intervention.

The final version of the checklist consists of a set of 17 questions; 12 applicable to individual studies and 5 applicable to a body of evidence. The content of this checklist is based on systematic searches of the literature, a three-round Delphi procedure, and a pilot test to assess the usability and any challenges associated with the use of the checklist. In view of the rigorous development process, we believe that the checklist is comprehensive, relevant and acceptable as well as useable.

The checklist presented in this paper differs in several aspects from previously reported checklists [15, 17]. First, our checklist has a wider scope of applicability. Previously published checklists focus on the appraisal of subgroup analyses in trials, whereas our checklist also covers other study types such as non-randomized studies and systematic reviews. As moderators and predictors can be identified through a broad range of study designs and analyses, this is of special value in areas where trials are not feasible.

Second, our checklist aims to help researchers to assess the overall relevance of moderators or predictors within clinical decision making or health technology assessments. It therefore contains criteria aimed at assessing transferability and comparison of effects across a body of evidence and thus helps to obtain an overall judgment not offered by previous checklists. All criteria in the checklist are further described in a user’s guide, facilitating use by those who lack specialized knowledge of predictor or moderator analyses. Additional file 3 contains an overview of the most important differences between our checklist and previous checklists.

A generic difficulty in developing and using appraisal checklists lies in distinguishing between the quality of a study and the quality of its reporting. Inevitably, a good standard of reporting is required if one is to properly appraise a moderator or predictor. For this reason, our checklist also includes criteria that relate to reporting quality (e.g. mentioning pre-specification of hypotheses). Also, there may be a relation between good reporting quality and good methodological quality. For instance, if researchers are aware of the fact that predefined hypothesis testing is important, they are likely to report this in their article. Therefore, criteria that are not properly reported are more likely to be of poor quality.

It should be noted that for several criteria within our checklist, no general agreement currently exists on what constitutes best practice. For instance, different statistical methods exist for subgroup identification, [52–54] but these are difficult to assess when researchers are not familiar with them. Although we aimed to provide guidance on these issues, it was difficult to provide strict criteria or specific cut-off points. For this reason, we recommend that any appraisal should involve a team with complementary skills, including clinicians as well as methodologists.

One limitation of the methods employed in our study lies in the use of the RAND Appropriateness Method for the selection of criteria. This means that the appropriateness of any individual criterion is dependent on the inclusion of all other criteria. In addition, the list of criteria is dynamic due to the ability of participants to suggest rewordings or additional criteria. Ideally, each change would require a reassessment of all criteria for appropriateness until agreement is reached. However, given the different backgrounds of the participants, valuations will not always converge. The appropriateness method rarely resulted in complete agreement on the exclusion of criteria, leaving room for the core research team to make final decisions. Ideally, the wording of criteria and the selection of criteria would have been split into separate phases. In spite of these limitations, there was broad agreement among the experts on the final version of the checklist, meaning that it appropriately reflects current views on how the validity of claims regarding prediction and moderation of treatment effects should be judged.

A second limitation is the fact that the Delphi panel was relatively small. Even though we believe that it comprised the relevant range of expertise across several countries, we may not have accommodated all available viewpoints. Although we performed a very extensive literature search, we may have missed articles, due to the diversity and variability of the terminology used in personalized, stratified and precision healthcare. [55–57] We do think the range of experts in our Delphi panel was able to compensate for any possibly missed relevant literature. Furthermore, the pilot test was relatively modest in the number of articles on which the checklist was piloted, so the future use of CHAMP will provide important additional insights regarding the usability of the checklist and the implications of its use for how we judge moderator and predictor findings.

## Conclusion

An appraisal checklist was created to help researchers formally appraise moderator or predictor analyses. The contents of the checklist were based on literature, three Delphi rounds and pilot testing. The use of such a checklist is relevant as moderator and predictor analyses are becoming increasingly common as the demand for personalized health care is growing. The CHAMP checklist expands upon existing tools, providing coverage and clarification for appraisal of randomized and non-randomized studies, as well as bodies of evidence. We tested its feasibility in an extensive pilot study. More experiences from different users and new developments in the future will allow a further refining and improvement of CHAMP.

## Additional files

Additional file 1: Contains the history of the criteria contained in the checklist and their valuation and transformation throughout the entire study. (XLSX 26 kb)

Additional file 2: Contains the product of this study, the CHAMP checklist, including packground information. (PDF 510 kb)

Additional file 3: Contains a table with the comparison of our checklist with those of Sun et al. and Pincus et al. (PDF 630 kb)

## Abbreviations

CHAMP: Checklist for the Appraisal of Moderators and Predictors
IPRAS: InterPercentile Range Adjusted for Symmetry
RAND: Research And Development

## References

[1] Egger M, Moons K, Fletcher C (2016). GetReal: from efficacy in clinicla trials to relative effectiveness in the real world.

[2] Yusuf S, Wittes J, Probstfield J, Tyroler HA (1991). Analysis and interpretation of treatment effects in subgroups of patients in randomized clinical trials. JAMA.

[3] Donegan S, Williams L, Dias S, Tudur-Smith C, Welton N (2015). Exploring treatment by covariate interactions using subgroup analysis and meta-regression in cochrane reviews: a review of recent practice. PLoS One.

[4] Kravitz RL, Duan N, Braslow J (2004). Evidence-based medicine, heterogeneity of treatment effects, and the trouble with averages. Milbank Q.

[5] Davidoff F (2009). Heterogeneity is not always noise: lessons from improvement. JAMA.

[6] Dettori JR, Norvell DC, Skelly AC, Chapman J (2011). Heterogeneity of treatment effects: from “how to treat” to “whom to treat”. Evid Based Spine-Care J.

[7] Debray TP, Moons KG, van Valkenhoef G, Efthimiou O, Hummel N, Groenwold RH, Reitsma JB (2015). GetReal methods review G: get real in individual participant data (IPD) meta-analysis: a review of the methodology. Research synthesis methods.

[8] Hunter JE, Schmidt FL (1990). Methods of meta-analysis: correcting error and bias in research findings.

[9] Kent DM, Rothwell PM, Ioannidis JPA, Altman DG, Hayward RA (2010). Assessing and reporting heterogeneity in treatment effects in clinical trials: a proposal. Trials.

[10] Sacristán JA (2013). Patient-centered medicine and patient-oriented research: improving health outcomes for individual patients. BMC Med Inform Decis Mak.

[11] Baron RM, Kenny DA (1986). The moderator-mediator variable distinction in social psychological research: conceptual, strategic, and statistical considerations. J Pers Soc Psychol.

[12] Kraemer HC, Kiernan M, Essex M, Kupfer DJ (2008). How and why criteria defining moderators and mediators differ between the Baron & Kenny and MacArthur approaches. Health Psychol.

[13] Pocock SJ, Assmann SE, Enos LE, Kasten LE (2002). Subgroup analysis, covariate adjustment and baseline comparisons in clinical trial reporting: current practice and problems. Stat Med.

[14] Gabler NB, Duan N, Liao D, Elmore JG, Ganiats TG, Kravitz RL (2009). Dealing with heterogeneity of treatment effects: is the literature up to the challenge. Trials.

[15] Sun X, Briel M, Busse J, Akl E, You J, Mejza F, Bala M, Diaz-Granados N, Bassler D, Mertz D (2009). Subgroup analysis of trials is rarely easy (SATIRE): a study protocol for a systematic review to characterize the analysis, reporting, and claim of subgroup effects in randomized trials. Trials.

[16] Sun X, Briel M, Busse JW, You JJ, Akl EA, Mejza F, Bala MM, Bassler D, Mertz D, Diaz-Granados N (2012). Credibility of claims of subgroup effects in randomised controlled trials: systematic review. BMJ (Clinical research ed).

[17] Pincus T, Miles C, Froud R, Underwood M, Carnes D, Taylor S (2011). Methodological criteria for the assessment of moderators in systematic reviews of randomised controlled trials: a consensus study. BMC Med Res Methodol.

[18] McCormack LA, Treiman K, Rupert D, Williams-Piehota P, Nadler E, Arora NK, Lawrence W, Street RL (2011). Measuring patient-centered communication in cancer care: a literature review and the development of a systematic approach. Social science & medicine (1982).

[19] Moons KGM, de Groot JAH, Bouwmeester W, Vergouwe Y, Mallett S, Altman DG, Reitsma JB, Collins GS (2014). Critical appraisal and data extraction for systematic reviews of prediction Modelling studies: the CHARMS checklist. PLoS Med.

[20] Hua H, Burke DL, Crowther MJ, Ensor J, Tudur Smith C, Riley RD (2017). One-stage individual participant data meta-analysis models: estimation of treatment-covariate interactions must avoid ecological bias by separating out within-trial and across-trial information. Stat Med.

[21] Developing and evaluating complex interventions: new guidance [http://www.mrc.ac.uk/documents/pdf/complex-interventions-guidance/].

[22] Sanderson S, Tatt ID, Higgins JP (2007). Tools for assessing quality and susceptibility to bias in observational studies in epidemiology: a systematic review and annotated bibliography. Int J Epidemiol.

[23] Deeks JJ, Dinnes J, D'Amico R, Sowden AJ, Sakarovitch C, Song F, Petticrew M, Altman DG: Evaluating non-randomised intervention studies. *Health Technol Assess* 2003, **7**(27):iii-x, 1–173. 10.3310/hta7270 14499048

[24] Jüni P, Witschi A, Bloch R, Egger M (1999). The hazards of scoring the quality of clinical trials for meta-analysis. JAMA.

[25] Katrak P, Bialocerkowski AE, Massy-Westropp N, Kumar S, Grimmer KA (2004). A systematic review of the content of critical appraisal tools. BMC Med Res Methodol.

[26] Fitch K, Bernstein SJ, Aguilar MD, Burnand B, LaCalle JR: The RAND/UCLA appropriateness method user's manual. Santa Monica, CA, RAND; 2001.

[27] Brereton L, Wahlster P, Lysdahl KB, Mozygemba K, Burns J, Chilcott JB, Ward S, Brönneke JB, Tummers M, Van Hoorn R (2016). Integrated assessment of home based palliative care with and without reinforced caregiver support: ‘a demonstration of INTEGRATE-HTA methodological guidances’.

[28] Milberg A, Strang P (2004). Exploring comprehensibility and manageability in palliative home care: an interview study of dying cancer patients’ informal carers. Psycho-Oncology.

[29] Mercadante S, Valle A, Porzio G, Costanzo BV, Fusco F, Aielli F, Adile C, Fara B, Casuccio A (2011). How do cancer patients receiving palliative care at home die? A descriptive study. J Pain Symptom Manag.

[30] Cartoni C, Niscola P, Breccia M, Brunetti G, D'Elia GM, Giovannini M, Romani C, Scaramucci L, Tendas A, Cupelli L (2009). Hemorrhagic complications in patients with advanced hematological malignancies followed at home: an Italian experience. Leukemia & lymphoma.

[31] Chvetzoff G, Garnier M, Perol D, Devaux Y, Lancry L, Chvetzoff R, Chalencon J, Philip T (2005). Factors predicting home death for terminally ill cancer patients receiving hospital-based home care: the Lyon comprehensive cancer center experience. J Pain Symptom Manag.

[32] Abdelnour-Mallet M, Verschueren A, Guy N, Soriani MH, Chalbi M, Gordon P, Salachas F, Bruneteau G, le Forestier N, Lenglet T (2011). Safety of home parenteral nutrition in patients with amyotrophic lateral sclerosis: a French national survey. Amyotroph Lateral Scler.

[33] Ruggeri E, Agostini F, Fettucciari L, Giannantonio M, Pironi L, Pannuti F (2013). Home artificial nutrition in advanced cancer patients. Tumori.

[34] Santarpia L, Alfonsi L, Pasanisi F, De Caprio C, Scalfi L, Contaldo F (2006). Predictive factors of survival in patients with peritoneal carcinomatosis on home parenteral nutrition. Nutrition.

[35] Chakravorty I, Chahal K, Austin G (2011). A pilot study of the impact of high-frequency chest wall oscillation in chronic obstructive pulmonary disease patients with mucus hypersecretion. Int J Chron Obstruct Pulmon Dis.

[36] Cano NJ, Pichard C, Roth H, Court-Fortune I, Cynober L, Gerard-Boncompain M, Cuvelier A, Laaban JP, Melchior JC, Raphael JC (2004). C-reactive protein and body mass index predict outcome in end-stage respiratory failure. Chest.

[37] Zuo ML, Yue WS, Yip T, Ng F, Lam KF, Yiu KH, Lui SL, Tse HF, Siu CW, Lo WK (2013). Prevalence of and associations with reduced exercise capacity in peritoneal dialysis patients. Amer J Kidney Dis.

[38] Krishnasamy R, Badve SV, Hawley CM, McDonald SP, Boudville N, Brown FG, Polkinghorne KR, Bannister KM, Wiggins KJ, Clayton P (2013). Daily variation in death in patients treated by long-term dialysis: comparison of in-center hemodialysis to peritoneal and home hemodialysis. Am J Kidney Dis.

[39] Pauly RP, Maximova K, Coppens J, Asad RA, Pierratos A, Komenda P, Copland M, Nesrallah GE, Levin A, Chery A (2010). Patient and technique survival among a Canadian multicenter nocturnal home hemodialysis cohort. Clin J Am Soc Nephrol.

[40] Sharma SK, Chaurasia RK, Sijapati MJ, Thapa L, Ghimire M, Shrestha H, Acharya A, Khanal B (2010). Peritonitis in continuous ambulatory peritoneal dialysis. JNMA; J Nepal Med Assoc.

[41] Li PK, Law MC, Chow KM, Leung CB, Kwan BC, Chung KY, Szeto CC (2007). Good patient and technique survival in elderly patients on continuous ambulatory peritoneal dialysis. Perit Dial Int.

[42] Gonzalez-Perez JG, Vale L, Stearns SC, Wordsworth S (2005). Hemodialysis for end-stage renal disease: a cost-effectiveness analysis of treatment-options. Int J Technol Assess Health Care.

[43] Woodd SL, Grosskurth H, Levin J, Amuron B, Namara G, Birunghi J, Coutinho A, Jaffar S (2014). Home-based versus clinic-based care for patients starting antiretroviral therapy with low CD4(+) cell counts: findings from a cluster-randomized trial. AIDS (London, England).

[44] Weschules DJ (2005). Tolerability of the compound ABHR in hospice patients. J Palliat Med.

[45] Morita T, Sato K, Miyashita M, Akiyama M, Kato M, Kawagoe S, Kinoshita H, Shirahige Y, Yamakawa S, Yamada M (2013). Exploring the perceived changes and the reasons why expected outcomes were not obtained in individual levels in a successful regional palliative care intervention trial: an analysis for interpretations. Support Care Cancer.

[46] Alonso-Babarro A, Astray-Mochales J, Dominguez-Berjon F, Genova-Maleras R, Bruera E, Diaz-Mayordomo A, Centeno Cortes C (2013). The association between in-patient death, utilization of hospital resources and availability of palliative home care for cancer patients. Palliat Med.

[47] Wiese CH, Morgenthal HC, Bartels UE, Vossen-Wellmann A, Graf BM, Hanekop GG (2010). Post-mortal bereavement of family caregivers in Germany: a prospective interview-based investigation. Wien Klin Wochenschr.

[48] Ahlner-Elmqvist M, Jordhoy MS, Jannert M, Fayers P, Kaasa S (2004). Place of death: hospital-based advanced home care versus conventional care. A prospective study in palliative cancer care. Palliat Med.

[49] Bengoechea I, Gutierrez SG, Vrotsou K, Onaindia MJ, Lopez JM (2010). Opioid use at the end of life and survival in a Hospital at Home unit. J Palliat Med.

[50] Sun X, Briel M, Walter SD, Guyatt GH. Is a subgroup effect believable? Updating criteria to evaluate the credibility of subgroup analyses. BMJ. 2010;340 10.1136/bmj.c117 20354011

[51] Gagnier J, Morgenstern H, Altman D, Berlin J, Chang S, McCulloch P, Sun X, Moher D (2013). Group ftAACHC: consensus-based recommendations for investigating clinical heterogeneity in systematic reviews. BMC Med Res Methodol.

[52] Su X, Zhou T, Yan X, Fan J, Yang S: Interaction trees with censored survival data. *The international journal of biostatistics* 2008, 4(1): Article 2. 10.2202/1557-4679.1071 PMC2835451 20231911

[53] Foster JC, Taylor JM, Ruberg SJ (2011). Subgroup identification from randomized clinical trial data. Stat Med.

[54] Berger JO, Wang X, Shen L (2014). A Bayesian approach to subgroup identification. J Biopharm Stat.

[55] Janes H, Pepe MS, Bossuyt PM, Barlow WE (2011). Measuring the performance of markers for guiding treatment decisions. Ann Intern Med.

[56] Mandrekar SJ, Sargent DJ (2010). Predictive biomarker validation in practice: lessons from real trials. Clinical trials.

[57] Simon R (2008). Development and validation of biomarker classifiers for treatment selection. J Stat Plan Inference.

