# Supplementary material for: The development of CHAMP: a checklist for the appraisal of moderators and predictors
Source: BMC Med Res Methodol. 2017 Dec 21;17:173. doi: 10.1186/s12874-017-0451-0 (PMC5740883; doi:10.1186/s12874-017-0451-0)
Supplement: Supplementary file 2 — Contains the product of this study, the CHAMP checklist, including packground information. (PDF 510 kb) [file 12874_2017_451_MOESM2_ESM.pdf]

CHAMP: CHecklist for the Appraisal of Moderators and Predictors

About the checklist

In this document, a CHecklist for the Appraisal of Moderators and Predictors (CHAMP) is presented. Information on treatment effects can be used to build prediction models or identify subgroups which may respond differently to treatments. CHAMP aims to appraise evidence and assess relevance of the evidence for heath technology assessments (HTAs) or guidelines. The document has three sections: an introduction, the appraisal checklist itself, and a background section on how the checklist may be used.

CHAMP is designed to provide users with a structured way of looking at a set of key quality and relevancy indicators. The checklist is intended to be applicable for various study types such as (randomised) intervention trials, observational studies and systematic reviews. The background information provided at the end of this document is intended to help users answering the items and interpreting the consequences of negative answers for the overall judgment of credibility and relevance of treatment moderation or prediction.

CHAMP requires that overall study quality (unrelated to treatment moderation or prediction) is assessed, using appropriate appraisal tools. The user of the checklist is asked to report the conclusion from this appraisal before proceeding with the appraisal of claims regarding moderation or prediction. The checklist itself consists of twelve items grouped under the headings of design, analysis, results, and transferability of results. An additional set of five items can be used to appraise claims regarding moderation or prediction of treatment effects based on a body of evidence.

Moderators versus predictors: definition

For moderators and predictors of treatment effects we used the definitions by (Baron & Kenny, 1986). According to their definitions, moderators and predictors are variables such as patient characteristics that influence the effect of a treatment. The difference between moderators and predictors is that a moderator influences the effect of a specific treatment (e.g. the protective effect of aspirin is moderated by gender), while a predictor influences outcomes regardless of any treatment (e.g. old age predicts higher probability of infections). A predictor will show the same effect over all treatment arms within subgroups, while a moderator will show a different effect in each arm. Because of this difference, moderators should be tested using statistics such as interaction tests (e.g. a treatment by characteristic-interaction in a regression model), while predictors are not tested for interaction. With the exception of the interaction term, the analysis of moderator and predictor analyses adhere largely to the same quality criteria. Quantitative moderators can only be retrieved from (preferably randomised) intervention studies or systematic reviews as the investigation of moderators should include the evaluation of subgroup differences between a intervention and a control group). Predictors on the other hand may be retrieved from many different kinds of studies, including observational studies.

Appraisal of overall study quality

A large number of critical appraisal tools is available to assess overall study quality. Widely used appraisal tools for the various study designs include:

- **Systematic review / meta-analysis:** AMSTAR checklist (http://amstar.ca); CASP Systematic Review Checklist[1]; Cochrane's Risk Of Bias[2] tool (used on the individual studies included in the review).If the systematic review is based on individual patient data (IPD), the guidance by Tierney et al. may be useful (doi:10.1371/journal.pmed.1001855). Lastly, there is the CHARMS checklist for systematic reviews of prediction modelling studies by Moons et al. (doi:10.1371/journal.pmed.1001744).
- **Randomised controlled trial:** Cochrane's Risk of Bias tool[2]; CASP Randomised Controlled Trial[1] checklist.
- **Cohort study:** CASP Cohort Study Checklist[1] ; Newcastle-Ottawa scale (http://www.ohri.ca/programs/clinical_epidemiology/nosgen.pdf).
- **Cross-sectional / descriptive study**: Cross sectional appraisal tool (https://reache.files.wordpress.com/2010/03/cross-sectional-appraisal-tool.pdf).
- **Case-control study:** CASP checklist for case-control studies[1]; Newcastle-Ottawa scale (http://www.ohri.ca/programs/clinical_epidemiology/nosgen.pdf).
- **Prognostic study**: Quality In Prognosis Studies (QUIPS) (http://www.biomedcentral.com/content/supplementary/1546-0096-12-19-S1.pdf)

Further appraisal tools have been identified by Katrak et al., 2004. Please indicate the tool that was used to critically appraise the overall study quality, and the major findings below.

Critical appraisal tool used for assessing overall study validity *(if any)*

Overall study validity – *Outcome of the used appraisal tool or argumentation on the quality of the study*

Is the overall study quality of a level that findings related to moderators and predictors are likely to be of sufficient quality?

☐ Yes -> please continue appraisal

☐ No -> do not continue appraisal

☐ Don't know -> please continue appraisal, but mind possible bias

[1] *CASP appraisal tools are available from http://www.casp-uk.net/#!checklists/cb36*
[2] http://ohg.cochrane.org/sites/ohg.cochrane.org/files/uploads/Risk%20of%20bias%20assessment%20tool.pdf

Appraisal of moderators and predictors for treatment effects

This checklist aims to appraise study findings concerning moderators or predictors. It may be that multiple candidate moderators or predictors of treatment effect have been explored within a single study. In such cases, credibility or relevance may differ across these factors, depending on how they were measured, strength of association, etc. It is up to the user to decide whether conclusions apply to all candidate factors that were examined or to subsets only (and this checklist should be applied for each set separately).

Answering: choose 'yes' if the item applies, 'no' if it does not. Use 'don't know' to indicate that you do not consider yourself sufficiently qualified to judge the relevant item, or that the article does not report sufficient information to answer the question. Use 'Not applicable' if the item does not apply to the moderator or predictor being appraised.

	Yes	No	Don't know	Not applicable
**Design**				
1. A priori plausibility: was there sufficient empirical or theoretical support for the moderator or predictor that was examined?	☐	☐	☐	☐
2. Was the moderator or predictor specified a priori?	☐	☐	☐	☐
3. Was the moderator or predictor variable measured before the allocation or start of the intervention?	☐	☐	☐	☐
4. Was measurement of the moderator or predictor reliable and valid in the target population?	☐	☐	☐	☐
**Analysis**				
5. In case of a moderator, was an interaction test used?	☐	☐	☐	☐
6. Was a limited number of moderators and predictors tested?	☐	☐	☐	☐
7. Was sample size adequate for the moderator or predictor analysis?	☐	☐	☐	☐
**Results**				
8. Were results presented for all candidate moderators or predictors that were examined?	☐	☐	☐	☐
9. Did statistical tests or confidence intervals indicate that observed moderator or predictor effects were unlikely to be merely due to chance variation?	☐	☐	☐	☐
10. Was the moderator or predictor effect consistent with related moderators or predictors, or across related outcomes measured within the study?	☐	☐	☐	☐
**Transferability**				
11. Were the setting and study population comparable to the setting and population in which the information would be used?	☐	☐	☐	☐
12. Is the moderator or predictor effect clinically important?	☐	☐	☐	☐

	Yes	No	Don't know
**Overall judgement**<br>Considering your conclusions regarding overall quality and items 1-12, would you say that claims regarding moderation or prediction of treatment outcomes are sufficiently substantiated and sufficiently relevant to take into account when making recommendations for treatment decisions?	☐	☐	☐

Please clarify your main arguments to support your conclusion:

Appraisal of moderators and predictors for treatment effects in a body of evidence

Specific candidate moderators or predictors of treatment effect may have been explored in multiple studies. Appraisal of such a body of evidence is important as some aspects of appraisal, such as comparison of effects between studies or relevance of a moderator or predictor effect, become apparent only after moderator or predicting findings have been collected from multiple studies.

For such cases, items 10-12 are repeated here (as items 13-15) as they apply to the summarised or pooled effect and may be answered differently as compared to individual studies. For example, smaller studies may find apparently large and clinically important effects, but when pooled with other, larger studies, the effect may no longer be statistically or clinically significant. These items can also be used to appraise studies within meta-analyses, not meta-analyses themselves.

**Body of evidence**	Yes	No	Don't know	Not applicable
13. Was the moderator or predictor effect consistent with related moderators or predictors, or across related outcomes measured <u>between the studies</u>?	☐	☐	☐	☐
14. Were the setting and study population comparable to the setting and population in which the information would be used?	☐	☐	☐	☐
15. Is the moderator or predictor effect clinically important?	☐	☐	☐	☐
16. Was the moderator or predictor effect reasonably homogenous across studies?	☐	☐	☐	☐
17. Was the moderator or predictor measured similarly across the included studies, or was an adequate conversion performed?	☐	☐	☐	☐

**Overall judgment**	Yes	No	Don't know
Considering the answers for individually appraised studies as well as items 10-14, would you say that claims regarding moderation or prediction of treatment outcomes are sufficiently substantiated and sufficiently relevant to take into account when making recommendations for treatment decisions?	☐	☐	☐

Please clarify your main arguments to support your conclusion:

Background on the appraisal items

Below, for each item a brief rationale is presented, considerations that you may want to take into account, and a brief discussion of possible implications when a study does not seem to meet the relevant criterion.

Item 1 A priori plausibility: was there sufficient empirical or theoretical support for the moderator or predictor that was examined?

Basically, this item asks about independent empirical or theoretical support for the candidate moderator(s) or predictor(s) of treatment effect. In case of such support, it is less likely that the moderator or predictor effect was a spurious result.

Consider: Whether the authors provided a plausible (biological) working mechanism. Preferably, this is based on experimental studies and generally accepted as a possible biological pathway. In case of a meta-analysis, a-priori is more difficult to define; assuming study collection was systematic, one could look at the amount of studies finding or suggesting the proposed moderator or predictor.

Implications: A moderator or predictor effect is more likely to be a false-positive finding if there is no underlying theory on how the effect could influence the outcome. However, the observed effect may have been caused by other mechanisms, yet unknown.

Item 2 Was the moderator or predictor specified a priori?

A moderator or predictor effect should preferably be specified a priori. A finding is less likely to be a chance finding if the moderator or predictor effect (direction and/or size) was hypothesised before the start of the study.

Consider: Whether the hypothesised effects and analyses were specified in a previously published study protocol, or whether they were explained by studies referenced in the paper, or whether authors explicitly stated that candidate factors were pre-specified. Any analysis to estimate the statistical power of the study for specified moderator/predictor effects also indicates that the moderator or predictor was pre-specified.

Implications: A moderator or predictor effect is more likely to be false-positive if its analysis was not pre-specified. In such cases, findings should be considered exploratory, in need of further verification

Item 3 Was the moderator or predictor variable measured before the allocation or start of the intervention?

The earlier variables are measured in a study, the less prone their measurements are to bias (e.g. measurements errors correlated to the treatment arm the patients were allocated to).

Consider: Whether the variable was measured before allocation or start of the intervention, for instance because the variable was used for stratified allocation or because it is explicitly stated. This does not apply to variables that are unlikely to be affected by treatment.

Implications: If the variable is straightforward to measure without bias and insensitive to treatment (e.g. age, gender) there is little reason for concern. Otherwise, two problems may arise [1] the measured effect may be a mediator, the variable explains part of the outcome because it is part of the causal relationship between treatment and outcome. In this case the variable cannot be used to stratify treatments. [2] Different types of measurement bias could have occurred. If the study was double-blinded this is less likely.

Item 4 Was measurement of the moderator or predictor reliable and valid in the target population?

Unreliable or invalid measurements of predictors or moderators can result in either under- or overestimation of the moderator or predictor effect. If the moderator or the predictor is not measured using a reliable and valid method, the subgroup effect may be underestimated and the main treatment effect may be overestimated, or vice versa.

Consider: Whether the measurement method is reliable and valid in the target population as evidenced by pilot testing and/or existing publications on the measurement method. Also consider, if the population used to estimate or discover moderator or predictor effects is not the same as the overall population, what implications in terms of bias such a selection may have introduced. For instance, part of a population may have been excluded for moderator analysis due to missing data. If these missing values are in any way systematic, bias is introduced.

Implications: Credibility of the effect (size) is compromised, in proportion to doubts regarding reliability or validity of the measurement.

Item 5 In case of a moderator, was an interaction test used?

In the literature, the terms ‘moderator’ and ‘predictor’ are occasionally used interchangeably (or other terms are used to describe their effects, such as effect modifier, determinant or interaction effect). Hence, it is important that the user first identifies whether the effect that is being appraised is actually a moderator, a predictor, both (a factor can have both moderation and prediction effects), or other

effect (e.g. mediating effect or main effect). Please be aware that a subgroup difference should not be considered a moderator effect unless this difference is explicitly tested.

Consider: Whether an interaction-test was used (a treatment by moderator interaction) or a similar statistical test to determine the significance of subgroup differences over different treatment groups. Note that not all moderator effects are multiplicative; additive moderator effects may appear similar to predictor effects and can only be distinguished by determining whether the factor can be used to explain outcome differences between subgroups receiving a treatment. Also consider whether the effect can be considered a main effect instead (i.e. direct theoretical or statistical association with treatment).

Implications: If no interaction-test was used, or the results of such test were not statistically significant, the observed effect (if any) should be considered a predictor, not a moderator. If the moderator is associated/correlated with the treatment, it is a mediator.

Item 6 Was a limited number of moderators and predictors tested?

There are two reasons for keeping the number of moderators and predictors tested at a minimum: The probability of finding false-positive effects due to chance (related to alpha-level), and the risk of overfitting of (regression) models.

Consider: Consider the total number of moderators and predictors tested in a study. There are no firm criteria to determine what number of tests can still be considered adequate. These problems are (at least partially) related to the amount of tests performed in relation to study size. There are some rules of thumb relating to multivariate analysis: Some sources state that 20 subjects per moderator is the bare minimum (Pincus et al., 2011), other rules of thumb range between 2-20 cases per regression parameter, and up to 50 per parameter in the case of stepwise regression (Austin & Steyerberg, 2015; Voorhis & Morgan, 2007). The minimal number of cases per parameter increases when effect sizes are expected to be small, when there are substantial measurement errors or when data are skewed (Voorhis & Morgan, 2007). For univariate analysis, specific statistical methods may be used to perform or correct for multiple testing (e.g. Bonferroni correction) to enhance reproducibility of study findings and increase the number of tested moderators or predictors that can be reasonably estimated. Statistical expertise may be required to appraise such cases.

Implications: Depending on the p-value that is still considered significant (often set at 0.05) or the size of the confidence interval (often set at 95%), a certain number of hypotheses are expected to be significant based on chance alone (in the case of a p-value of 0.05, this is 5% of all tested hypotheses). Hence, if more tests are performed, the more likely that a finding is false-positive.

Item 7 Was sample size adequate for the moderator or predictor analysis?

Without adequate sample size, the odds of false-negative moderator or predictor effects is increased (i.e. moderators or predictors may be missed). This, in turn, may result in underestimation of other effects if such findings are used as a basis for multivariate analysis. The user may wish to perform post-hoc power analysis or look at similar studies that do provide a power analysis if none is provided in the study. There is no agreed upon best practice in sample size calculation for moderator or predictor analysis, so some leniency here should be employed.

Consider: Consider whether a power analysis (ad hoc or post hoc) was performed or other consideration on study size were described; did the researchers succeed in including and following up the requisite number of patients? Also, consider measures such as model fit and error sizes. Please keep in mind that there is a difference between a statistical significant effect and a significant clinically meaningful effect. A significant effect that will not affect clinical decision making is more likely to be found if the sample size is large.

Implications: If the sample size of a study was inadequate, effects that did not reach significance may have actually been significant effects if a larger study was performed. Hence, observed effects to be not statistically significant cannot be dismissed. If interaction terms are not significant and study size was too small, there may yet be a moderator effect.

Item 8 Were results presented for all candidate moderators or predictors that were examined?

Conceivably, more candidate moderators or predictors were investigated, but only those for which significant associations were found are being reported (selective reporting or reporting bias). This may be established by examining published study protocols. Additionally, to properly interpret moderator effects, predictor effects of the same variable (if any) should be investigated and presented as well.

Consider: Any clues that there were more moderators or predictors tested, such as statements like ‘all other variables were not significant’ or ‘results were corrected for baseline characteristics’ without explicit statement of the results. Consider variables that were mentioned in the protocol (if any) but not reported, or moderators or predictors that were much more likely to be researched than those reported in the study. On the other hand, if a study reports insignificant moderators/predictors, this may be considered an indication that the researchers were comprehensive in reporting moderation or prediction effects. Presented results of moderator or predictor analysis should show at least the interaction coefficients, confidence intervals and/or test statistics.

Implications: If it is likely that more moderators or predictors were investigated than reported, it is possible that results were selectively reported. This increases the likelihood that reported effects were chance findings.

Item 9 Did statistical tests or confidence intervals indicate that observed moderator or predictor effects were unlikely to be merely due to chance variation?

Statistical tests help distinguish chance findings from real findings. Although the results of these tests do not prove that a moderator or predictor effect is real or not, they do add to the evidence that an observed effect is likely to be true.

Consider: Whether the (pooled) moderator or predictor effect shows statistical significance (e.g. confidence interval excluding the null hypothesis, or p-value lower than the value considered to be significant; usually 0.05 or lower in the case of many tests or the utilisation of a Bonferroni or other correction). If a correction for multiple testing was used, consider the validity of this method as well as its assumptions (see also items 5 and 6).

Implications: Smaller intervals and better significance add to the credibility of the observed effect. Insignificance of moderator (interaction-term) effects may indicate that there is another effect (e.g. predictor-effect) or that study size was inadequate. Insignificance of a predictor-effect may indicate the predictor-effect does not exist or that the study was too small.

Item 10 & 13 Was the moderator or predictor effect consistent with related moderators or predictors, or across related outcomes measured within the study [or between studies]?

Consistence between moderator or predictor effects adds to the credibility of the results. Inconsistency between findings may suggest chance findings or incorrect assumptions in theories or analysis (e.g. correlations between two regression models parameters may result in two oppositely directed effects). ‘Related’ means sharing a (pathophysiological) pathway or related characteristics (e.g. employment and income). The stronger such measures are related, the more consistent the results should be. For systematic reviews or a body of evidence, this item does not involve the comparison of the same moderator or predictor effect across different studies, but the comparison of different moderator or predictor effects.

Consider: Whether multiple related moderators or predictors show the same effects (variation in effect size may apply) for the same outcome; whether a single moderator or predictor shows similar effect across different related outcomes (e.g. survival and incidence of infection).

Implications: If related moderators or outcomes show similar effects, this contributes to the credibility of individual findings. If contradictory effects are found, other effects may underlie the observed moderator or predictor effects, or observed effects apply only under specific conditions.

Item 11 & 14 Were the setting and study population comparable to the setting and population in which the information would be used?

The identification of moderators and predictors helps increasing the transferability of findings, but it remains important to determine whether findings from a study can be validly applied to the target setting.

Consider: Whether target and sampled population are similar for moderators and predictors that have been tested (e.g. same range of age), as well as similar on moderators and predictors that have not been tested (geographical location, socio-economical-status, support from others, etc.).

Implications: Credibility of findings is compromised when they have to be extrapolated to different settings or populations. Moderator or predictor effects may behave differently in other settings (being moderated themselves), for instance because of underlying or related moderator or predictor effects, or practical differences in the treatment applied.

Item 12 & 15 Is the moderator or predictor effect clinically important?

A clinically important moderator or predictor is one that has a considerable effect (i.e. larger than any measurement error as well as sufficient size) and one that is likely to be able to be implemented in practice. For example, ethical, legal or practical issues may prohibit the use of moderators or predictors in clinical practice or assessment, thus reducing its usefulness. Unless authors make explicit statements on the clinical importance of a moderator or predictor effect, specific expertise may be needed to address this item.

Differences between subgroups (predictor or moderator effects) should always be interpreted with caution, even if they are based on formal tests. Explicit presentation of findings in different subgroups may help in the correct interpretation of the relevance of the results. The difference between subgroups can be presented as a mean difference (with standard deviation), standardized mean differences or Cohen's d.

Consider: Whether the reported group moderator or predictor effect is clinically relevant. Also consider whether confidence intervals or statistical significance were reported; as without these measures clinical importance is more difficult to estimate. Confidence intervals add to the interpretability of effects and add to the validity of the estimation of effects when used in models. In the case of individual patient data meta analysis (IPDMA), please disregard individual studies effects as they may show only very small or unstable effects due to their limited size. For IPDMA, pooled effect size should be considered only.

Furthermore, see whether the effects that were found, in relation to their practical implementation, may be considered of benefit. This needs to be related to any difficulties one may encounter when implementing the moderator or predictor. For instance, ethical problems may be overcome if the

effect is very large. If the authors made statements on clinical importance, be critical and try to assess whether their arguments stand firm.

Implications: For individual studies the implications may be small. It is better to reconsider clinical importance in view of all the evidence available on the moderator or predictor. If the finding is from a large study or body of evidence, the implication may be that the observed effect, although credible, may not be practicable in daily care.

Item 16 Was the moderator or predictor effect reasonably homogenous across studies?

Moderator and predictor effects should be homogenous across different studies. Effect sizes are likely to vary to some degree, but the greater the differences the less likely the moderator or predictor effect is a true effect. This is especially true if the effects over studies are contradictory (e.g. one study showing a protective effect, while another showing a harmful effect of a moderator). If low agreement between studies is found, it is important to determine possible causes: differences in study populations, study designs, methods of analysis, or actually different moderator or predictor effects?

Consider: Whether the moderator or predictor effects are approximately equally sized (i.e. the conclusion of the moderator or predictor effect would not change if studies are excluded) across studies and point in the same direction. In the case of individual patient data meta analysis (IPDMA), please disregard individual studies effects as they may show only very small or unstable effects due to their limited size. For IPDMA, pooled effect size should be considered only.

Implications: Differences in the size or direction of effects impact the credibility of the findings. This impact can be quantified by performing a meta-analysis. If larger differences are found, or even worse, differences in direction of an effect, the credibility of findings is clearly compromised.
It may also be possible that differences between studies can be explained. If this is the case, one could say that there are moderated moderators or predictors. Establishing the effects of such an extra level of moderation should, however, be regarded as an additional analysis. That is, all considerations in this checklist apply to that moderator as well. It may be quite challenging to estimate the effects of these factors to a useful degree without performing further research.

Item 17 Was the moderator or predictor measured similarly across the included studies, or was there an adequate conversion performed?

One problem that moderators and predictor share with many outcomes is that they can be measured differently between studies. Another problem with moderators and predictors is that if moderators or predictors are investigated in a multivariate

method, they can greatly influence the effect of the other moderators or predictors in the same model.

Consider: Whether the moderator or predictor effect is measured using a similar instrument (e.g. the same questionnaire, scale or other tool). If this is not the case, consider whether conversions are possible. Also consider, if moderators or predictors are assessed in multivariate analysis, whether a similar set of moderators/predictors/other factors is taken into account.

Implications: If measurements of moderators or predictors are not comparable, it is difficult to determine a pooled effect, to determine usefulness of results or credibility of a body of evidence. If that is the case, one should adhere to the tools that are used in the target setting.
If the moderator or predictor was included in multivariate models with different sets of other included factors, those other factors may greatly influence the observed effect of a moderator, and thus the comparability of the moderator or predictor across studies. Furthermore, it may be more difficult to determine the effect size if it cannot be established which other moderator or predictors need to be taken into account.

References

Austin, P. C., & Steyerberg, E. W. (2015). The number of subjects per variable required in linear regression analyses. *J Clin Epidemiol, 68*(6), 627-636. doi: 10.1016/j.jclinepi.2014.12.014

Baron, R. M., & Kenny, D. A. (1986). The moderator-mediator variable distinction in social psychological research: conceptual, strategic, and statistical considerations. *Journal of Personality and Social Psychology, 51*, 1173-1182.

Katrak, P., Bialocerkowski, A. E., Massy-Westropp, N., Kumar, S., & Grimmer, K. A. (2004). A systematic review of the content of critical appraisal tools. *BMC Med Res Methodol, 4*, 22. doi: 10.1186/1471-2288-4-22

Pincus, T., Miles, C., Froud, R., Underwood, M., Carnes, D., & Taylor, S. (2011). Methodological criteria for the assessment of moderators in systematic reviews of randomised controlled trials: a consensus study. *BMC Medical Research Methodology, 11*(1), 14.

Voorhis, C. R. W. V., & Morgan, B. L. (2007). Understanding Power and Rules of Thumb for Determining Sample Sizes. *Tutorials in Quantitative Methods for Psychology*.
