# Supplementary material for: The development of CHAMP: a checklist for the appraisal of moderators and predictors
Source: BMC Med Res Methodol. 2017 Dec 21;17:173. doi: 10.1186/s12874-017-0451-0 (PMC5740883; doi:10.1186/s12874-017-0451-0)
Supplement: Supplementary file 3 — Contains a table with the comparison of our checklist with those of Sun et al. and Pincus et al. (PDF 630 kb) [file 12874_2017_451_MOESM3_ESM.pdf]

	CHAMP	SATIRE checklist [10] / SUN 2010 [19]	Pincus [12]
Aim of tool	to assess individual studies or body of evidence on moderator or predictor findings	to assess subgroup quality as part of a systematic review	to assess moderator findings for inclusion in systematic reviews
Aimed at (M)oderators or (P)redictors	M and P	M and P (referred to as 'subgroup effects')	M only
Development method	new checklist	extension of existing set of 7 criteria by Oxman and Guyatt [23]	new checklist
Source of criteria	literature & Delphi panel	existing checklist & expert opinion	literature & Delphi panel
Target study types	Quantitative or quantitative studies	randomized trials (for inclusion in systematic reviews)	randomized trials (for inclusion in systematic reviews)
Type of questions	closed (polar)	open (polar)	open (polar)
Remarks	contains two lists:<br>1 for rating individual studies<br>1 for body of evidence	SATIRE checklist: Not presented as standalone checklist; developed for their review specifically. Sun 2010: similar to SATIRE checklist but presented as checklist	contains two lists:<br>1 for exploratory meta-analyses<br>1 for confirmatory meta-analyses
Primary output	overall verdict and answers to individual items	unspecified; answers to individual questions	unspecified; answers to individual questions
Background information on item usage	included	included for n=4 new items (Sun 2010 only). Remainder only through references	Included in publication, backup up by references but limited in usability as a standalone document
Total number of items	13 (individual studies)<br>15 (body of evidence)	11	3 (exploratory)<br>5 (confirmatory)
Prespecification of hypothesis	yes	yes (also directional)	yes (only in confirmatory list)
Prespecificaiton of analysis	yes	no	yes (only in confirmatory list)
Interaction test usage	yes	yes	yes
Credibility of findings	(indirect through biol. Rationale and effect size)	(indirect through biol. rationale)	yes (only in confirmatory list, theory or evidence-based plausibility)
Consistency (within and between studies)	yes (between studies only in body-of-evidence list)	yes	no
Transferability of findings	yes	no	no
Was factor measured at	yes	yes (only refers to randomisation)	yes (only refers to randomisation)

baseline or before randomisation			
Biological rationale	yes	yes (‘indirect evidence’)	yes (only in confirmatory list)
Independence other effects	no	yes	no
Effect within versus between studies	no	yes	no
Limited to a small number of tests	yes	yes	no
Effect size	yes (clinical importance)	yes	no
Measurement quality	yes	no	yes
Sample size	yes	no	no
Completeness of tests reported	yes	no	no
Statistical reporting	yes	no	no

Table 1: comparison of CHAMP checklist (this study) with two checklists by Sun et al.[10, 19](both are similar, hence compared simultaneously) and a moderator checklist by Pincus et al. [12].
